# Cold Atmospheric Pressure Plasma Treatment of Maize Grains—Induction of Growth, Enzyme Activities and Heat Shock Proteins

**DOI:** 10.3390/ijms22168509

**Published:** 2021-08-07

**Authors:** Ľudmila Holubová, Renáta Švubová, Ľudmila Slováková, Boris Bokor, Valéria Chobotová Kročková, Ján Renčko, Filip Uhrin, Veronika Medvecká, Anna Zahoranová, Eliška Gálová

**Affiliations:** 1 Department of Genetics, Faculty of Natural Sciences, Comenius University in Bratislava, Mlynská dolina, Ilkovičova 6, 842 15 Bratislava, Slovakia; f.uhrin@gmail.com (F.U.); eliska.galova@uniba.sk (E.G.); 2 Department of Plant Physiology, Faculty of Natural Sciences, Comenius University in Bratislava, Mlynská dolina, Ilkovičova 6, 842 15 Bratislava, Slovakia; ludmila.slovakova@uniba.sk (Ľ.S.); boris.bokor@gmail.com (B.B.); krockovavaleria@gmail.com (V.C.K.); rencko.jan@gmail.com (J.R.); 3 Comenius University Science Park, Comenius University in Bratislava, 841 04 Bratislava, Slovakia; 4 Department of Experimental Physics, Faculty of Mathematics, Physics and Informatics, Comenius University in Bratislava, Mlynská dolina F1, 842 48 Bratislava, Slovakia; medvecka3@uniba.sk (V.M.); zahoranova1@uniba.sk (A.Z.)

**Keywords:** CAPP, DCSBD, DNA damage, germination, glucanase, heat shock proteins, non-thermal plasma, maize, protease

## Abstract

*Zea mays* L. is one of the most produced crops, and there are still parts of the world where maize is the basic staple food. To improve agriculture, mankind always looks for new, better methods of growing crops, especially in the current changing climatic conditions. Cold atmospheric pressure plasma (CAPP) has already showed its potential to enhance the culturing of crops, but it still needs more research for safe implementation into agriculture. In this work, it was shown that short CAPP treatment of maize grains had a positive effect on the vitality of grains and young seedlings, which may be connected to stimulation of antioxidant and lytic enzyme activities by short CAPP treatment. However, the prolonged treatment had a negative impact on the germination, growth, and production indexes. CAPP treatment caused the increased expression of genes for heat shock proteins HSP101 and HSP70 in the first two days after sowing. Using comet assay it was observed that shorter treatment times (30–120 s) did not cause DNA damage. Surface diagnostics of plasma-treated grains showed that plasma increases the hydrophilicity of the surface but does not damage the chemical bonds on the surface.

## 1. Introduction

Maize *(Zea mays* L.) is one of the oldest crops that humanity has domesticated. It belongs to the most produced cereal crops, together with wheat and rice, and its production is estimated to grow steadily in the next decade. It is an important staple food in many parts of the world, and maize for human consumption is estimated to rise, especially in Sub-Saharan Africa. Besides food production for humans, there is a strong demand for bioethanol and animal feed and other maize products such as corn starch or syrup [1]. It is thus essential to ensure effective and sustainable methods of growing maize, where new technologies like plasma agriculture could have a place.

Plasma is ionized gas containing various reactive, neutral, and charged particles. Non-thermal, low-temperature or “cold plasma” means that heavy particles—ions and neutrals—remain near room temperature, and electrons reach high temperature due to their high mobility [2,3,4]. Therefore, a remarkable characteristic of cold plasma is the low gas temperature, but also the presence of reactive components, UV radiation (mainly in nitrogen or N_2_-containing gases such as air), and high-energy electrons. Cold atmospheric pressure plasma (CAPP) is very suitable for practical use. Its production does not require a low-pressure vacuum device. The exact composition of non-thermal plasma (NTP) depends on the type of plasma source and the working gas used. For example, CAPP generated in different ratios of O_2_:N_2_ by Diffuse Coplanar Surface Barrier Discharge (DCSBD) analyzed by Fourier Transform Infrared spectroscopy (FTIR) and optical emission spectroscopy showed the presence of oxygen and nitrogen species (NO, NO_2_, N_2_O, N_2_O_5_, HNO_3_) and excited molecules (NO* or N_2_*, N_2_^+^*) responsible for producing radiation in the UV region by deexcitation. Additionally, a relatively high amount of ozone is produced in an oxygen-rich mixture [5].

The use of non-thermal plasma in agriculture is a promising concept since many studies state that plasma treatment of seeds can enhance germination and growth parameters of seedlings [6,7,8,9] and sterilize the surface of seeds [10,11,12], which is vital for cereal crops, since diseases may be detrimental to the economy and threaten food supplies [13]. It was observed that the enhancement of germination is related to the changes on the seed surface after the NTP treatment [14,15,16]. Reactive particles in NTP interact with the surface, usually making it more hydrophilic [17,18]. NTP does not cause any surface damage; only with a significantly prolonged treatment time fine cracks can appear, which depends on the properties of the surface. The reactive plasma species interact with the surface, penetrate it and induce secondary elementary reactions [6,19]. These changes can result in quicker water absorption during imbibition and quicker induction of germination [14,15,16,20]. Besides the faster water uptake, the activity of enzymes, which mobilize the seed reserves, is essential. The increase in activity of proteases and glucanases, which was observed in seedlings from plasma-treated seeds, is also connected to the enhancement of germination and growth [20,21].

There are many reactive oxygen and nitrogen species (RONS) in the NTP. On the one hand, RONS contained in NTP (e.g., ˙O_2_^−^, ˙OH, NO) may play a role in the breaking of dormancy and enhancing seeds’ germination [22]. On the other hand, however, NTP treatment of seeds or seedlings may cause oxidative or nitrosative stress. Indeed, Billah et al. [23] observed increased amounts of H_2_O_2_ in roots and leaves of *Vigna mungo* L., especially for longer exposure times, after the treatment of seeds with NTP. Some authors noticed an increase in the activity of antioxidant enzymes, such as superoxide dismutase, catalase, or peroxidases [22,24,25]. However, the results regarding the activity of antioxidant enzymes are a bit ambiguous. For example, Henselová et al. [26] did not observe significant changes in the activity of these enzymes in maize seedlings from NTP-treated grains, and Tong et al. [27] noticed the increase in the activity only for catalase in one experimental group, and even a decline in the activity of superoxide dismutase (SOD) in several groups.

Differences in the effects of NTP treatment between publications may be caused by the variety of plasma devices. For example, DCSBD plasma source is CAPP, specified by the high surface and volume power density (appr. 2.4 W·cm^−2^, 80 W·cm^−3^, resp.), which leads to the short treatment times (up to 20–50 s), suitable for practical applications [28]. On the other hand, Puač et al. [29] had to use treatment times in a range of tens of minutes with their low-pressure plasma generated by a radio-frequency capacitively coupled plasma reactor at lower powers (50–200 W). To compare the effect of NTP on different species, the plasma source should be the same. However, pre-sowing plasma treatment is also species-specific. For example, Štěpánová et al. [30] compared the CAPP treatment of pepper and cucumber seeds and found that both species have different optimal treatment times. This suggests that it is important to study the effect of NTP treatment on various plant species.

In plants, there are several general defenses against various stresses. Besides antioxidants, heat shock proteins (HSPs) are among the first to respond. As molecular chaperones, their role is to refold, disaggregate, or send to degradation misfolded proteins and prevent aggregation. The activity of HSPs and heat shock factors (HSFs) is also important in priming or acquired tolerance to stress [31,32]. For example, Lämke et al. [33] stated that HSFA2 (Heat shock factor A2) is essential for sustained thermotolerance in *Arabidopsis*. A similar result was observed by Charng et al. [34]. HSFA4A (Heat shock factor A4A) was found to be important for salt and oxidative stress tolerance in *Arabidopsis* [35]. HSP70s are the most abundant HSPs in cells, and besides physiological functions, they are necessary under various stresses. The increase of HSP70 protein was observed, for example, in *Lemna minor* as a response to exposure to heavy metals [36].

This paper studied the influence of CAPP treatment of maize grains on the germination and growth parameters and activities of lytic and antioxidant enzymes. We verified physical and chemical changes on the maize grains surface due to plasma treatment in different working gases. We also monitored the DNA damage caused by this treatment and looked at the expression of some heat shock protein genes to find if they are involved in the response of maize to the CAPP treatment.

## 2. Results and Discussion

### 2.1. ATR-FTIR

In Attenuated Total Reflectance-Fourier Transform Infrared spectroscopy (ATR-FTIR) spectra acquired from the surface of maize grains, we can identify the characteristic chemical groups attributed to lipids, carbohydrates, and proteins. In 3000–2800 cm^−1^ the significant peaks attributed to symmetric and asymmetric stretching of methyl and methylene (CH_3_ and CH_2_) group can be visible, originating primarily from lipids [37,38]. Additionally, the deformation vibrations of C–H bonds occur at 1460–1400, 1370, and 1240 cm^−1^ in “the fingerprint” region [39]. Carbohydrates are presented by a typical glycosidic bond at around 1020 cm^−1^ and other bonds in region 1200–800 cm^−1^ [40]. Three distinct groups represent proteins: amide I (stretching vibration of C=O) at 1640 cm^−1^, amide II (stretching of C–N and deformation of N–H) in 1550–1520 cm^−1^ and amide III (vibrations of N–H, C–N, C–C bonds) at about 1240 cm^−1^. In addition, the weak band of amide A (N–H stretching vibration) can be found in a spectra of proteins at about 3200 cm^−1^, but it is overlapped by a strong, wide band of OH stretching in 3600–3000 cm^−1^ [41].

**Figure 1:**
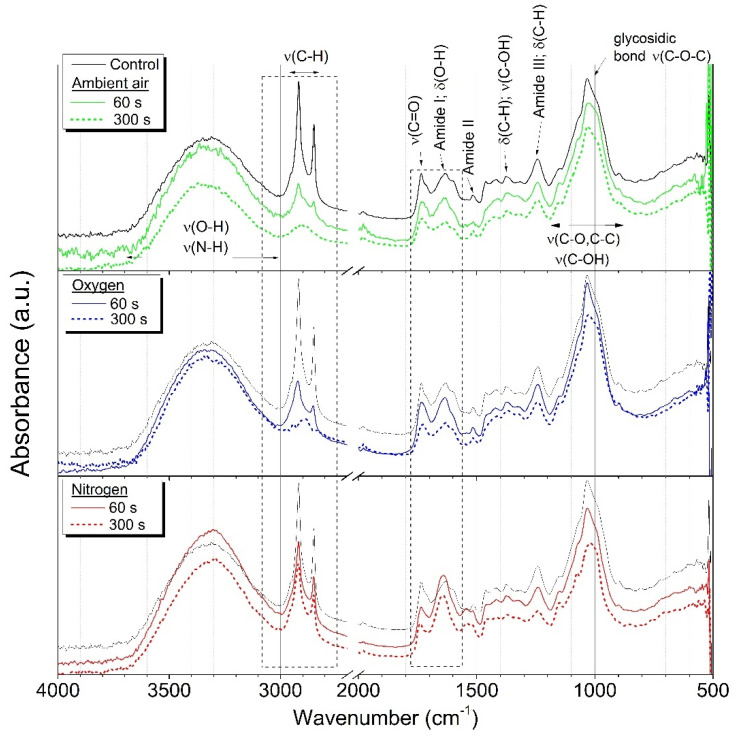
Attenuated Total Reflectance-Fourier Transform Infrared spectroscopy (ATR-FTIR) spectra of maize grains untreated and after plasma treatment in ambient air, oxygen and nitrogen, treatment time 60 s, 300 s, at input power 400 W.

The changes due to the plasma treatment of samples can be noticed in two regions; 3000–2800 cm^−1^, where we can observe the decrease of C–H bonds, and in interval 1800–1550 cm^−1^, in which the carbonyl (C=O) group is typical. This effect is considerable on samples treated by air and oxygen plasma. A decrease in chemical groups typical for lipids indicate the activation of the maize surface by oxidation and removal of the top lipid layer (Figure 1).

### 2.2. Wettability

**Figure 2:**
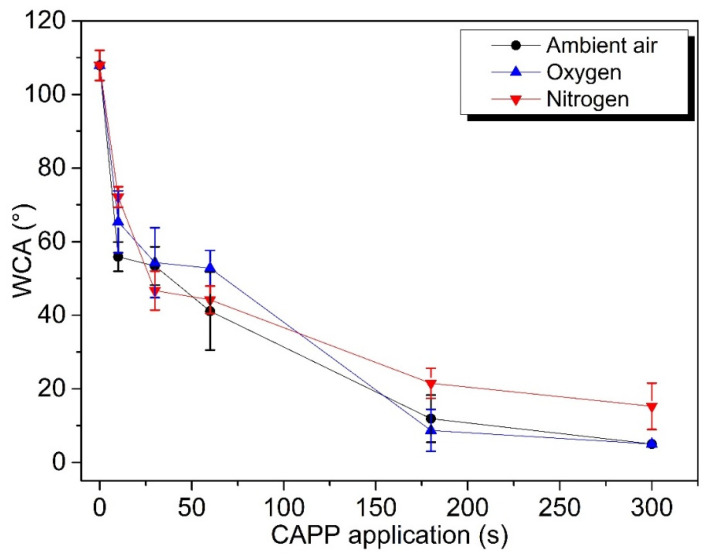
Effect of plasma exposure time on the water contact angle (WCA) measured on maize grains, samples treated by ambient air, oxygen, and nitrogen Diffuse Coplanar Surface Barrier Discharge (DCSBD) plasma at atmospheric pressure, the input power was 400 W. Values are the mean ± SD, *n* = 10.

The water contact angle (WCA) measured on untreated samples was 107.9 ± 4.1°, thus, the surface is considered hydrophobic (WCA > 90°). After plasma treatment, the contact angle significantly decreased even at a short exposure time. After 30 s of treatment, the value of WCA was reduced approximately by 50% in all working gases. The value below <5°corresponds to the complete spreading out of the droplets, which was reached in air (A) and oxygen (O) plasma after 300 s of treatment. In nitrogen (N), the contact angles were higher after a longer treatment time (180 s and 300 s) (Figure 2). This effect is related to the different mechanism of plasma action on grains surface due to the different active particles generated in nitrogen (mainly reactive nitrogen species), where the absence of oxygen leads to low ability to remove lipids from the surface observed by ATR-FTIR analysis.

### 2.3. Physiological and Biochemical Parameters

Seed germination is the first and very critical period in the life cycle of each plant. The seeds must absorb water to activate their metabolism, and this hydration kinetics takes place in three phases [42,43]. First, seeds absorb water through physical mechanisms and this phase is independent of the metabolic activity of the seeds so that it occurs equally well in living and dead seeds. However, dead or damaged seeds can absorb more water than live seeds because the turgor pressure in live seeds acts against hydration [44]. Imbibition of seeds initiates a sequence of events that results in the mobilization of reserves to the embryo, elongation and division of cells and subsequent protrusion of the radicle through surrounding layers [45]. It is known that plasma treatment can affect seed germination at the initial imbibition stage [46]. Our results show that the application of CAPP had a positive effect on imbibition in all studied variants (except the A30 variant).

**Figure 3:**
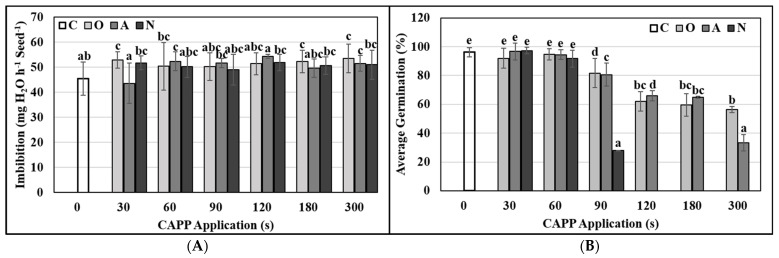
Imbibition (**A**) and germination (**B**) (%) of maize grains after Cold Atmospheric Pressure Plasma (CAPP) treatment. Variants: C—control/untreated maize grains; O30–O300: maize grains treated with plasma generated in an oxygen atmosphere for 30, 60, 90, 120, 180 or 300 s; A30–A300: maize grains treated with plasma generated in ambient air for 30, 60, 90, 120, 180 or 300 s; N30–N300: maize grains treated with plasma generated in a nitrogen atmosphere for 30, 60, 90, 120, 180 or 300 s. Different letters indicate significant difference at *p*-value < 0.05, bars are means of ten experimental runs (one run represents 50 grains per variant, *n* = 500) ± SD to LSD ANOVA test.

Statistically, significantly more water was taken up by maize grains treated with CAPP which was generated in an oxygen atmosphere for 30, 180 and 300 s, and in CAPP which was generated in ambient air for 60 and 300 seconds (Figure 3A). Many authors claim that plasma application can partially disrupt/modify seed/grain coating layers. Thus reactive particles can penetrate deeper and directly interact with storage tissues or with the embryo [6,47]. Low-temperature plasma significantly modifies the surface of seeds and grains, thereby altering their affinity for water [48,49]. Zahoranová et al. [50] found out that application of CAPP for 10 seconds on maize grains (cv. Ronaldinio) resulted in the reduction of the water contact angle by 50%. They did not notice any damage to the grain surface even after prolonged exposure to CAPP, but they observed higher grain hydrophilicity and higher water uptake. We suppose that it may be partly caused by the surface properties of maize grains, especially by their hardness and the gentle diffusion character of DCSBD plasma. These findings agree with our results, and this effect can be explained by the increasing concentration of polar groups containing oxygen and nitrogen on the surface of treated maize grains. Our results are consistent with the observations of other authors [17,51].

As the maize grains (cv. Ronaldinio) used in this study had approximately 95% germination, we did not notice an increase in the germination percentage due to CAPP application. However, the germination rate at the low exposure times (in all used working gases) remained statistically comparable to the untreated control. On the other hand, the quicker germination of maize grains after exposure to the magnetic field might be due to increased activities of germination related enzymes, fast hydration of membranes and greater molecular mobility of water [52]. Despite the increased imbibition, we observed a significant adverse effect on the germination of maize grains with prolonged exposure to CAPP (90 or more seconds in all used working gases). Doses of 120 or more seconds of CAPP generated in a nitrogen atmosphere completely inhibited germination (Figure 3B). Using FTIR Spectroscopy, it was confirmed that the generation of plasma in a nitrogen and ambient air atmosphere produces ultraviolet (UV) radiation [5], which is harmful in high doses (according to the WHO). Ultraviolet radiation is a part of the light spectrum and penetrates relatively deep through the seed coat. Nitrogen plasma produces more intensive UV radiation than ambient air plasma, which could be the reason of germination inhibition of grains treated by nitrogen plasma at 120 s or more. High doses of CAPP (90 or more seconds in all used working gases) also had a negative impact on growth and production indexes. The grains had a reduced germination potential (%), germination index (%) and vitality/vigor index (%). Young seedlings also had a negatively affected vitality/vigor index (%) and were generally shorter (seedling length index in %). Our results are also supported by the findings of Shao et al. [53], who stated that extended plasma application on maize grains decreased germination and vigor of young plants. In general, we can say that a shorter duration of CAPP (30 and 60 s) had a positive effect on the vitality of grains and young seedlings, which were longer compared to the untreated control (Table 1). Our statement is also confirmed by the results of Feng et al. [54] for the application of helium plasma, and Karmakar et al. [55] for the application of argon and oxygen plasma to maize grains. In the case of most variants with the lowest application time (from 30 to 60 s), we did not notice statistically significant differences in germination and growth of seedlings compared to the control. In O30 and N30 variants, the grain vitality index significantly increased. Prolonged exposure time caused stress and negatively influenced germination and growth parameters of maize.

**Figure 4:**
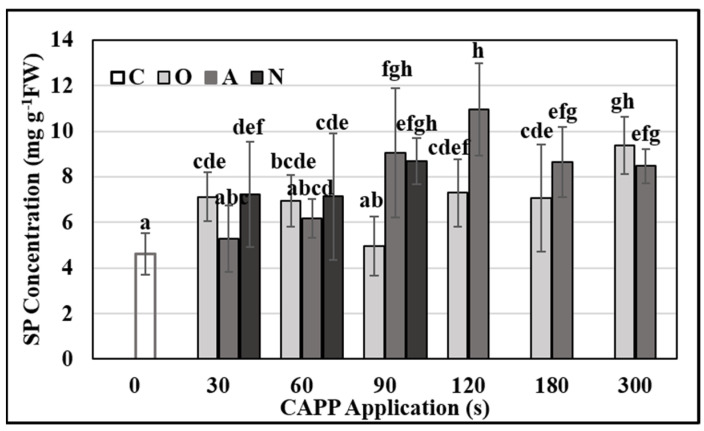
Total soluble protein (SP) concentration in 3-day-old maize seedlings after Cold Atmospheric Pressure Plasma (CAPP) treatment of grains. Variants: C—control/untreated maize grains; O30–O300: maize grains treated with plasma generated in an oxygen atmosphere for 30, 60, 90, 120, 180 or 300 s; A30–A300: maize grains treated with plasma generated in ambient air for 30, 60, 90, 120, 180 or 300 s; N30‒N300: maize grains treated with plasma generated in a nitrogen atmosphere for 30, 60, 90, 120, 180 or 300 s. In the case of N120–300 variants, the maize grains did not germinate, so they are not shown in the graph. Different letters indicate significant difference at *p*-value < 0.05, bars are means of ten experimental runs (one run represents 50 grains per variant; three 1.5 g mixed samples were analyzed per one experimental run and each variant for soluble protein concentration) ± SD according to LSD ANOVA test.

To find out the influence of plasma on biochemical mechanisms of plant germination, we also monitored the total soluble protein content and activities of lytic and antioxidant enzymes. The concentration of total soluble protein and the activity of lytic and antioxidant enzymes in three-day-old maize seedlings were affected by CAPP. In the case of the concentration of total soluble proteins (SP), we recorded an increase in all variants, and the trend changed depending on the working gas in which the CAPP was generated. Plasma generated in an atmosphere of oxygen and nitrogen caused an increase (statistically significant for O30, 60, 120, 180 and 300) in the concentration of total SP with increasing time of exposure to CAPP, compared to the untreated control. In the case of CAPP generated in ambient air, the concentration of total SP increased linearly until the treatment time of 120 s. After that there was a decrease, but even in 180 and 300 s treatment, the concentration of total SP was significantly higher (about two times higher) than in the untreated control (Figure 4). The increased concentration of proteins in the early stages of development after plasma treatment can be explained in two ways. Mobilization of storage proteins occurs in the early stages of germination. These storage proteins are mobilized by proteases and endopeptidases (especially cysteine proteases) and hydrolyzed to amino acids (AA), and subsequently deaminated to carboxylic acids (CA). Before proteins are hydrolyzed into AA and CA, the concentration of soluble proteins increases. These are then used as a source of energy in germination, growth, and development processes. The most studied was Papain-like cysteine protease which begins to be expressed in aleurone layer cells after imbibition [57]. The other possible explanation is the activation of the defense mechanisms of the young, germinated plant because in addition to mobilizing storage proteins, these cysteine proteases are known to play an important role in programmed cell death and also in the response of seedlings to biotic or abiotic stress factors [58]. In these processes, different stress proteins are synthesized. Chaperones maintain the correct conformation of proteins; ubiquitin designates damaged proteins, which are subsequently degraded by proteases.

Proteases are known to control signaling pathways in imbibed grains and plants. They play an important role, especially during germination, in the mobilization of storage proteins, such as free amino acids that can be used to build proteins and enzymes essential for embryo development [59]. However, these signaling pathways can also be disrupted by the action of highly reactive species that arise during NTP generation, which may explain the change in total soluble protein content in the germinated seedlings. The work of Henselová et al. [26] pointed to similar findings.

**Figure 5:**
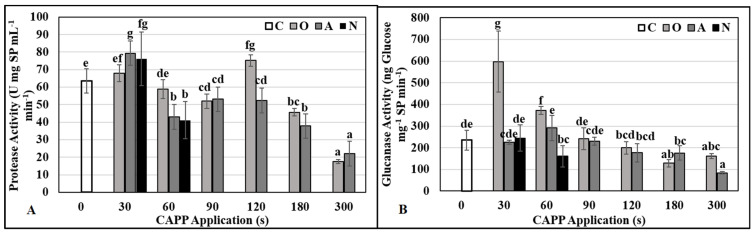
The activity of protease (**A**) and glucanase (**B**) in 3-day-old maize seedlings after Cold Atmospheric Pressure Plasma (CAPP) treatment of grains. Variants: C–control/untreated maize grains; O30–O300: maize grains treated with plasma generated in an oxygen atmosphere for 30, 60, 90, 120, 180 or 300 s; A30‒A300: maize grains treated with plasma generated in ambient air for 30, 60, 90, 120, 180 or 300 s; N30–N300: maize grains treated with plasma generated in a nitrogen atmosphere for 30, 60, 90, 120, 180 or 300 s. In the case of N90 variant seedlings lagged significantly in development and in N120–300 variants, the maize grains did not germinate, so they are not shown in the graph. Different letters indicate significant difference at *p*-value < 0.05, bars are means of ten experimental runs (one run represents 50 grains per variant; three 1.5 g mixed samples were analyzed per one experimental run and each variant for protease and glucanase activities) ± SD according to LSD ANOVA test.

Concentrations of total SP in individual variants correlated with protease activity Figure 5A). This means that in the case of the variant with high protease activity, the concentration of total SP decreased. We explain this by the fact that the high protease activity rapidly cleaved SP into amino acids and peptides, which cannot be detected by the methodology according to Bradford [60]. We assumed that the higher activity in three-day-old seedlings was due to proteases that should mobilize storage proteins at this stage of development, assemble proteins into the correct conformation and, in addition, break down damaged proteins. Glucanase activity increased rapidly only in the variants where the maize grains were treated for 30 and 60 s in CAPP generated in an oxygen atmosphere. In other variants, the activity of this enzyme was comparable to the untreated control (Figure 5B). During germination and early growth of seedlings, storage proteins are degraded and mobilized by proteases, with degradation of both subunits being similar but faster for β-conglycinin [61]. The storage proteins are cleaved into amino acids which, upon degradation to pyruvate, α-ketoglutarate, succinyl-CoA, fumarate and/or oxaloacetate, enter the Krebs cycle. Several authors agree that an appropriately chosen plasma application dose can have a positive effect on the activity of lytic enzymes and thus on the mobilization of storage substances in seeds and grains. Zhang et al. [25] reported that argon plasma positively affected soybean seed germination as well as the growth and development of young seedlings by regulating the level of demethylation of genes associated with energy metabolism. Acceleration of the germination process by positively influencing the activity of lytic enzymes (proteases, glucanases, amylases) is also presented in the works by Sadhu et al. [62] for mung beans, Švubová et al. [19] for pea seeds, Švubová et al. [20] for soybean, and Peťková et al. [21] for barley grains. The effective mobilization of storage substances and the increase of the soluble sugar and protein content in oilseed rape seedlings are documented by Ling et al. [63].

Low-temperature plasma generated in ambient air usually consists of reactive oxygen and nitrogen species such as NO_2_, N_2_O, NO, O_3_, HNO_3_, HNO_2_, CO_2_, ˙OH^−^, ˙O_2_^−^, ^1^O_2_. One of the possible mechanisms for plasma-induced stimulation of seed/grain germination is that the biochemical processes inside the seeds are activated by RONS [5]. Reactive oxygen and nitrogen species formed during plasma generation can act as signaling molecules and initiate a cascade of germination processes [64]. Emerging oxidative stress can lead to irreversible damage of lipids, proteins, membranes and DNA [65,66,67], but the extent of damage depends on the capacity of their antioxidant system. The removal of reactive oxygen species (ROS) from cells is ensured by various antioxidant mechanisms, including the enzymes SOD and peroxidases (POX). The superoxide anion is efficiently removed from the cells by the enzyme superoxide dismutase by dismutation to hydrogen peroxide. Guaiacol peroxidase (G-POX) prefers aromatic compounds such as guaiacol and pyrogallol as electron donors [68]. Because G-POX is active intracellularly (in the cytosol and vacuole), as well as extracellularly (in the cell wall), it is considered a key enzyme in the efficient removal of H_2_O_2_ [69].

**Figure 6:**
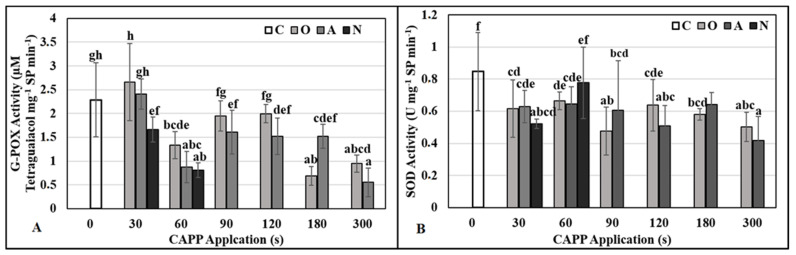
The activity of guaiacol peroxidase (G-POX) (**A**) and superoxide dismutase (SOD) (**B**) in 3-day-old maize seedlings after Cold Atmospheric Pressure Plasma (CAPP) treatment of grains. Variants: C—control/untreated maize grains; O30–O300: maize grains treated with plasma generated in an oxygen atmosphere for 30, 60, 90, 120, 180 or 300 s; A30–A300: maize grains treated with plasma generated in ambient air for 30, 60, 90, 120, 180 or 300 s; N30–N300: maize grains treated with plasma generated in a nitrogen atmosphere for 30, 60, 90, 120, 180 or 300 s. In the case of N90 variant seedlings lagged significantly in development and in N120–300 variants, the maize grains did not germinate, so they are not shown in the graph. Different letters indicate significant difference at *p*-value < 0.05, bars are means of ten experimental runs (one run represents 50 grains per variant; three 1.5 g mixed samples were analyzed per one experimental run and each variant for guaiacol peroxidase and superoxide dismutase activities) ± SD according to LSD ANOVA test.

After evaluating the activity of antioxidant enzymes, we can state that CAPP treatment did not increase oxidative stress in three-day-old maize seedlings. The activities of SOD in the treated variants were comparable to the untreated control (Figure 6B); the activity of G-POX (Figure 6A) increased slightly only in the variant O30.

Published works show that high application doses of cold plasma (180 or more seconds) negatively affect the activity of antioxidant enzymes. In pea seeds, the application of CAPP generated in the nitrogen atmosphere leads to a significant accumulation of superoxide radicals which indicates great oxidative stress [19]. This, in combination with the low activity of the enzyme SOD, probably causes the production of more toxic ROS and thus severe damage to biological membranes, killing of the embryo, and the inability of these seeds to germinate, as was the case in our experiments. This assumption is also supported by results obtained after applying plasma to soybean seeds [20]. This excessive oxidative stress, which increases in proportion to the plasma exposure time, can be explained by the production of ozone (O_3_) during the generation of plasma in an oxygen atmosphere, nitrogen oxides during generation of plasma in ambient air, and UV radiation during generation of plasma in a nitrogen atmosphere [5], which are harmful in high doses [70,71,72]. On the other hand, treatment with low-temperature plasma at shorter application doses (60 s) stimulated the activity of antioxidant enzymes. This mild stress, combined with a positive effect on lytic enzyme activity, had a stimulating effect not only on germination but also on the growth, development and overall vitality of young pea and soybean seedlings [19,20] and maize [26]. From the above, it can be stated that mild stress (increased intracellular ROS concentration and slightly increased activity of antioxidant enzymes) caused by short exposure to low-temperature plasma has a positive effect on the processes of germination, growth, and development of young seedlings of different plant species (pea, soybean). In the case of maize (hybrid Ronaldinio) the activity of antioxidant enzymes did not increase due to CAPP treatment.

### 2.4. Comet Assay

**Figure 7:**
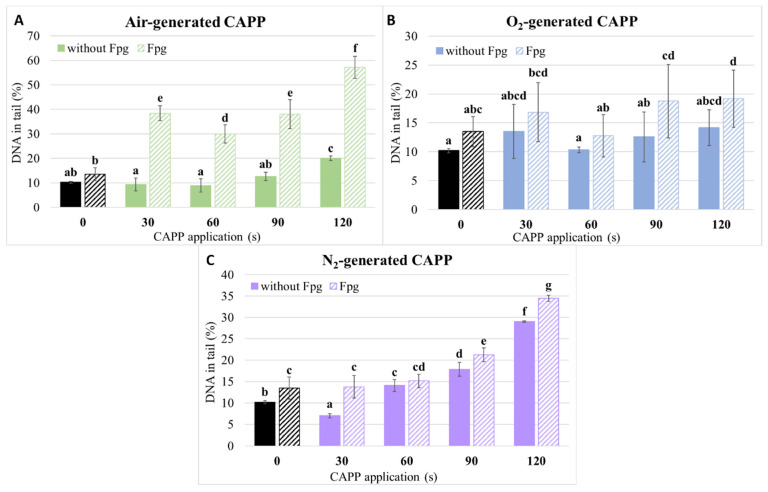
Level of DNA damage in 3-day-old maize seedlings detected by the neutral comet assay and its modification with enzyme formamidopyrimidine glycosylase (Fpg) after the treatment of maize grains with Cold Atmospheric Pressure Plasma (CAPP) generated in ambient air (**A**), oxygen (**B**) or nitrogen (**C**). 0 s—negative control (NC; no treatment); 30, 60, 90 and 120 s—treatment of grains with CAPP for 30–120 s; at least 100 nuclei were analyzed per slide. The data were analyzed using the statistical method LSD ANOVA and comparisons between the mean values were considered significant at *p* 0.05. Different letters indicate significant difference at *p*-value < 0.05, bars are the means of three experimental runs ± SD.

The neutral comet assay was used to assess the DNA damage caused by CAPP treatment. Based on the germination percentage, we decided to work only with four treatment times (30–120 s) since the average germination was already significantly decreased at 120 s. We found that the pre-sowing treatment of maize grains with CAPP generated in ambient air did not cause significant damage for shorter treatment times (30–90 s) and was similar to that of negative control (NC; 0 s). There was only a slight increase (10%) in DNA damage in the 120 s variant (Figure 7A). For plasma generated in oxygen, the DNA damage was similar to NC in all variants (30–120 s) (Figure 7B). For CAPP generated in nitrogen, however, there was a slight decrease for the 30 s treatment (compared to NC). Still, with increasing treatment time, the amount of DNA damage increased as well, up to 30% for the 120 s treatment, which was the highest amount of DNA damage caused by CAPP that we detected in this experiment (Figure 7C). The significant amount of oxidative DNA damage was detected mainly after the treatment with CAPP generated in ambient air, but only slight differences were present for CAPP generated in oxygen and nitrogen (Figure 7).

The DNA damage caused by CAPP treatment is usually not excessively high and increases with increasing treatment time, as is shown in other publications [5,19,20,21]. Our results agree with these works, as most of the variants caused only slight increases of DNA damage or were similar to NC, and the longest treatment times usually had the highest amount of DNA damage. Based on the germination of CAPP-treated maize grains, it seems that nitrogen-CAPP is the most toxic and, indeed, the amount of DNA damage was the highest, especially for the longest treatment times (90 and 120 s). In the study of Švubová et al. [19], the DNA damage was also the highest for nitrogen-generated CAPP in pea seedlings. However, in the study of Peťková et al. [21], the highest amount of DNA damage was recorded for CAPP generated in ambient air and oxygen in barley. These differences may be due to the different species used in the studies since the effect of plasma treatment is species-specific. Although three-day-old seedlings from grains treated with CAPP generated in ambient air and oxygen do not have high amounts of DNA breaks, they still contain some amount of oxidative DNA damage (more for air CAPP). Similar results were observed for barley [21]. This suggests that there is some level of oxidative stress in cells even a few days after the CAPP treatment. Overall, our results and other studies suggest that NTP treatment, especially for shorter treatment times, do not cause extensive DNA damage. Even though longer treatment times may cause a higher amount of damage, the cells are usually capable of repairing these lesions, provided that DNA repair mechanisms are not compromised by NTP treatment. However, in such cases, the germination and growth of seedlings would be compromised as well.

### 2.5. Expression of Heat Shock Proteins

As chaperones, heat shock proteins are essential for the cell not only in development and under normal conditions, but they are also crucial in stressful conditions, as stress often leads to various damages or denaturation of proteins [73]. We monitored the expression of *HSP101* and *HSP70* genes and heat shock factor gene *HSF17* during the 72 h after sowing by qPCR in samples where dry maize grains were treated with CAPP generated in ambient air.

**Figure 8:**
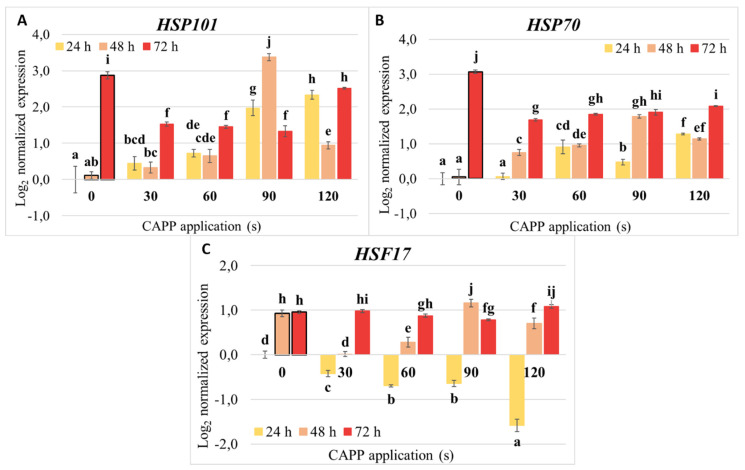
The relative expression of HSP101 (**A**), HSP70 (**B**) and HSF17 (**C**) in maize seedlings, which were treated with Cold Atmospheric Pressure Plasma (CAPP) generated in ambient air as grains for 0, 30, 60, 90 and 120 s, detected at 24, 48 and 72 h after sowing. Data are normalized to reference genes—tubulin β and elongation factor EF1α. The data were analyzed using the statistical method LSD ANOVA and comparisons between the mean values were considered significant at *p* 0.05. Different letters indicate significant differences between the treatments at *p*-value < 0.05 level.

We noticed that the expression of HSP101 and HSP70 reacted to the CAPP treatment of maize grains in a similar way. Their relative expression at 24 h increased with increasing CAPP treatment time (30–120 s) compared to the non-treated control (0 s). The expression in plasma-treated samples remained higher even at 48 h compared to the non-treated control. However, a different trend occurred at 72 h, where the highest expression was in a non-treated sample. At the same time, the CAPP treatment of grains resulted in a lower relative amount of transcripts (about half the amount of that in the control sample) (Figure 8A,B). The role of HSP101 is mainly the dissolving of aggregates, and together with HSP70 also refolding or removal of damaged proteins [74]. Therefore, it is no surprise that we detected a similar trend of expression in reaction to the CAPP treatment for both genes. The increase in the number of transcripts with increasing treatment times also suggests that longer exposure to CAPP causes more damage to proteins, which may also be the reason for the decrease in the activity of enzymes observed in this work. Some authors detected a sharp decrease in the number of *HSP101* transcripts in maize shortly after the germination [75,76]. However, we observed relatively high expression of *HSP101* and *HSP70* in the control sample at 72 h, and, on the contrary, lower expression in plasma-treated samples. A possible explanation is that it is related to the developmental processes of 72 h germination of young seedlings. Since the expression of both genes was influenced by CAPP treatment, it is possible that at 72 h, there is higher demand for chaperones in the non-treated control sample than in the CAPP-treated samples, where there was more of them in the past 48 h. Both HSP101 and HSP70 have essential roles during the heat, and other stresses [77,78] and our results suggest that they are also crucial for the reaction to CAPP treatment of seeds. Since their expression increased already at 24 h, the quick action of these two HSPs is needed in response to CAPP treatment as in the reaction to heat stress, where their expression also increased in the first 24 h according to Lämke et al. [33].

Heat shock factors are regulators of HSPs expression. Therefore, we included the HSF17 gene in the analysis of HSPs expression. A homologue of HSF17 in *Arabidopsis*, HSFA2, is an integral part of the maintenance of acquired thermotolerance in *Arabidopsis thaliana* [33,34], but it also increases the tolerance of *A. thaliana* to anoxia [32]. We observed that the relative amount of transcripts decreased with increasing CAPP treatment compared to non-treated control at 24 h after sowing. At 48 h, the amount of transcripts in CAPP-treated samples increased, but in shorter treatment times (30 and 60 s), it was still lower than in non-treated control. However, at 72 h, the amount of transcripts was already similar in all variants (Figure 8C). The decrease at 24 h suggests that in the reaction to the CAPP treatment the cells use other heat shock factors than HSF17 to regulate the HSPs expression. Iranbakhsh et al. [79] observed an increase in the expression of HSFA4A in wheat after the NTP treatment, which suggests that HSFA4A may have a role in the regulation of HSPs expression in response to the plasma treatment of plants.

## 3. Materials and Methods

### 3.1. Plant Material

Dried maize (*Zea mays* L. cv. Ronaldinio) grains used in these experiments were obtained from the Central Control and Testing Institute in Agriculture in Bratislava and KWS Semená s.r.o., Bratislava, Slovakia in 2017. Grains were stored in a fridge at 8 ± 2 °C in the dark.

### 3.2. Plasma Source and Treatment of Maize Grains

For the plasma treatment of the maize grains, the Diffuse Coplanar Surface Barrier Discharge (DCSBD) [80] was used as a source of cold atmospheric pressure plasma (CAPP). In our experiments, the plasma was generated in ambient air (A), oxygen (O) and nitrogen (N) at atmospheric pressure at the input power of 400 W. The grains were treated for 0 s (control), 30, 60, 90, 120, 180 and 300 s. The plasma treatment was performed in gas flow regime in a closed chamber with the experimental apparatus described in [5]. The gas flow rate was 3 L/min. The seeds were placed directly on a ceramic plate of DCSBD system, which was attached on the orbital shaker. The seeds were treated by plasma in dynamic regime at the frequency of 330 rpm to ensure the uniform treatment of grains. Since the area of the active plasma zone is (8 × 20) cm^2^, the number of treated seeds at once can be—according to the needs of the experiment and depending on seed size—about 200 pcs (for maize seeds). The DCSBD system was supplied by sinusoidal voltage with peak-to-peak amplitude of 20 kV and frequency 15 kHz.

### 3.3. ATR-FTIR

Attenuated Total Reflectance-Fourier Transform Infrared spectroscopy (ATR-FTIR) was used to determine the changes in specific chemical bonds due to the plasma treatment in different working gases on maize grains. Measurements were carried out using spectrometer Bruker Vector 22 with Pike MIRacle accessories (Pike Technologies, Madison, WI USA). The spectra were acquired in the range 4000–500 cm^−1^ during 20 scans for background/sample and resolution of 4 cm^−1^.

### 3.4. Wettability Properties

The wettability of the grain surface was evaluated by measurement of water contact angle (WCA) by Drop Shape Analyzer DSA30 (KRŰSS, Hamburg, Germany) with software DSA3. The contact angles of distilled water droplets with a volume of 2 µL were measured, and the average value was obtained from at least 10 measurements.

### 3.5. Imbibition, Germination and Growth Conditions

For one experimental run, dry maize grains (50 grains for each variant) were weighed on analytical scales and soaked in sterile distilled water for 4 h at room temperature. Imbibed grains were blotted dry, weighed again, and wrapped in wet sterile filter paper. Rolls were cultivated in dark conditions in an incubator at 24 ± 2 °C for 5 days. During cultivation, the number of germinated grains was counted, and material (seedlings without endosperm) for biochemical analyses was collected after 3 days. After 5 days, the length and weight of shoots and roots of young seedlings were measured. Obtained data were used to calculate germination (%), germination potential (%), germination index (%), grain vitality index (%), seedling vitality index (%), and seedling length index according to Abdul-Baki and Anderson [56].

### 3.6. Total Soluble Proteins Content

Samples (~1.5 g) were ground in liquid nitrogen with mortar and pestle and suspended in 50 mM Na-phosphate protein extraction buffer with 1 mM EDTA, pH 7.8. After 15 min centrifugation (12,000× *g*), the supernatant was used to determine protein concentration according to Bradford [60]. Total soluble proteins (SP) concentration was calculated as the amount of total proteins per gram of fresh matter from the calibration curve. We used Bovine Serum Albumin (BSA) as a protein standard.

### 3.7. Assay on Lytic Enzymes Assessment

The activity of β-1,3-Glucanase was assayed by measuring the rate of release of reducing sugar from laminarin (Sigma-Aldrich Co., Bratislava, Slovakia) as a substrate according to the methodology by Somogy [81] and Nelson [82]. Absorbance was measured by a spectrophotometer Jenway 6705 UV/Vis (Bibby Scientific Ltd., Essex, UK) at 660 nm. Total enzyme activity was calculated from the calibration curve where the glucose was used as a standard. Glucanase activity was expressed in nMol of glucose min^−1^ mg^−1^ of soluble proteins. Changes in the activity of protease in 3-day-old soybean seedlings were determined according to the methodology by Matušíková et al. [83]. The absorbance of the supernatant at 280 nm was measured by a spectrophotometer Jenway 6705 UV/Vis (Bibby Scientific Ltd., Essex, UK). One unit of proteolytic activity was defined as an increase of 0.001 in the absorbance at 280 nm per minute.

### 3.8. Assay on Antioxidant Enzymes Assessment

The activity of enzymes that detoxify ˙O_2_^−^ (superoxide dismutase (SOD), E.C.1.15.1.1) and H_2_O_2_ (guaiacol peroxidase (G-POX), E.C.1.11.1.7) was monitored. The activity of SOD was established according to Beauchamp and Fridowich [84] and the G-POX according to Frič and Fuchs [85]. One unit of SOD activity was the amount of proteins required to inhibit 50% of the initial reduction of Nitrotetrazolium Blue Chloride (NBT) under the light min^−1^ mg^−1^ of soluble proteins. Guaiacol peroxidase activity was expressed in µM of tetraguaiacol min^−1^ mg^−1^ of soluble proteins by molar extinction coefficient of tetraguaiacol 26.6 according to Chance and Maehly [86]. Chemicals were purchased from Sigma-Aldrich Co. (Bratislava, Slovakia).

### 3.9. Comet Assay

A neutral version of the comet assay was performed. The nuclei were isolated from 3-day-old maize seedlings using the slicing method with a sharp razor blade in 150 µL of ice-cold PBS (160 mM NaCl, 8 mM Na_2_HPO_4_, 4 mM NaH_2_PO_4_, 50 mM EDTA, pH 7) set on ice. For each sample, two roots were used, approximately one centimeter from the apex but excluding the root apex. The suspension of nuclei (100 µL) was mixed with 100 µL of 1% low melting point agarose (LMP), pipetted onto slides precoated with 1% normal melting point agarose, and covered with a coverslip. The nuclei fixed in LMP agarose on microscopic slides were lysed in cold high salt buffer (2.5 M NaCl, 100 mM EDTA, 10 mM Tris-HCl pH 7.5) for 20 min. Subsequently, electrophoresis was performed in 1x TBE (90 mM Tris-HCl pH 8.4, 90 mM Boric acid, 2 mM EDTA) at 1 V/cm at room temperature for 6 min. The slides were then dehydrated for 5 min in 75% ethanol and 5 min in 96% ethanol, left to dry and stored for subsequent evaluation. Nuclei were stained with 15 µL of 0.05 mM ethidium bromide for each mini gel on the slide. The stained nuclei were observed using fluorescence microscope OLYMPUS BX 51 with a green excitation filter UMWIG3 under 200× magnification and at least 100 nuclei per slide were evaluated by the visual scoring method [87].

The formamidopyrimidine (Fpg)-modified comet assay was based on a neutral comet assay described before with the Fpg additional steps according to Horváthová et al. [88]. Briefly, after the lysis step, the slides were washed three times for 5 min with an enzyme reaction buffer (40 mM HEPES, 0.5 mM EDTA, 0.1 M KCl, 0.2 mg/mL BSA, pH 8). Then, 0.2 U (40 µL) of the enzyme was added to each mini gel on the slide and the gels were then covered with a coverslip. The same amount of enzyme reaction buffer (instead of Fpg) was added to a parallel series of samples and covered with a coverslip. Both series were placed into a thermostat at 37 °C for 30 min. After the incubation, coverslips were removed, slides were washed 3 × 5 min in 1x TBE and the protocol continued with the step of electrophoresis as in the standard neutral comet assay.

As a negative control (NC), seedlings not pre-treated with CAPP or any other agent were used.

### 3.10. RT-PCR

All the experiments from RNA extraction to real-time PCR were performed by available commercial kits according to the manufacturer’s instructions. RNA was isolated from maize seedlings that were treated with CAPP generated in ambient air as grains, at 24, 48 and 72 h after sowing. For each sample, 2–3 seedlings without endosperm were used. Seedlings were immediately frozen in liquid nitrogen and ground with mortar and pestle. Total RNA was isolated using Spectrum Plant Total RNA Kit (Sigma-Aldrich, Bratislava, Slovakia). The RNA samples were treated with DNase I enzyme, purified and concentrated using RNA Clean & Concentrator™—5 columns (BioRad, Prague, Czech Republic). The concentration and purity of total RNA were measured with spectrophotometer Nanodrop 1000 (ThermoFisher Scientific, Prague, Czech Republic). The RNA integrity was checked by denaturing agarose gel electrophoresis [89]. Using iScript cDNA Synthesis Kit (BioRad, Prague, Czech Republic), the cDNA was synthesized by reverse transcription of 1 µg of total RNA. cDNA samples were used to assess the relative expression of chosen genes using qPCR. Specific transcripts were amplified using Luna^®^ Universal qPCR Master Mix (New England Biolabs, Ipswich, MA, USA) in thermocycler LightCyclerII 480 (Roche, Rotkreuz, Switzerland) with the set of primers listed in Table 2.

As reference genes, β-tubulin and elongation factor EF1α were used with the set of primers listed in Table 3.

Primers were designed using the online tool Primer-BLAST (http://www.ncbi.nlm.nih.gov/tools/primer-blast/; accessed on 4 November 2020) from sequences in the NCBI database.

Fluorescent quantification of products in individual samples took place during polymerization using SYBR/FAM filter. The specificity of PCR products was confirmed by agarose gel electrophoresis and melt curve analysis, which was included at the end of each run of the qPCR reaction. The sequencing of PCR products was performed by the Sanger method at the Department of Molecular Biology (Faculty of Natural Sciences, Comenius University). Sequences were analyzed using the program Sequencing Analysis software (version 5.4, Applied Biosystems, Waltham, MA, USA) and verified by the Blast bioinformatic tool (http://blast.ncbi.nlm.nih.gov/Blast.cgi; accessed on 12 January 2021).

### 3.11. Statistical Analysis

The data were analyzed using Microsoft Excel (Microsoft Office 2013, Redmond, WA, USA) and Statgraphic Centurion 19 (Statgraphics Technologies, Inc., The Plains, Virginia). Treatment effects were investigated by means of ANOVA single-step multiple comparisons using LSD test, and comparisons between the mean values were considered significant at *p* < 0.05. All experimental data in this work are from at least three independent experiments.

Relative change in gene expression was estimated according to Taylor et al. [90] including gene amplification efficiencies. Statistical analysis (ANOVA and LSD test) of gene expression data was based on the log_2_ transformed normalized expression per sample.

## 4. Conclusions

Cold atmospheric pressure plasma has a potential to be used in agriculture for seed treatment for more environmentally friendly production of various crops. Until then, it is necessary to study its effects on various plants to gain a holistic view and to be able to design optimal treatment conditions for the desired outcomes.

The presented work focused on the effects of CAPP treatment of maize grains. We found that after the treatment, the wettability of grain surface increased and also the amount of water taken up by the grains during imbibition was higher. The surface diagnostics using ATR-FTIR did not show damage of the surface chemical bonds of the grains after plasma treatment. The CAPP generated by DCSBD plasma source is characterized by high volume power density, so short plasma treatment times are enough to achieve a substantial improvement of seed germination and the vitality of grains and young seedlings. However, higher doses of CAPP (90 or more seconds) had more of a negative impact on growth and production indexes. Specifically, higher doses of CAPP generated in nitrogen completely inhibited germination. The increased amount of soluble proteins was observed after the plasma treatment which can be caused by mobilization of storage proteins by proteases. The activities of antioxidant enzymes in variants treated with air and oxygen plasma were lower or comparable to the negative control, suggesting that the oxidative stress in three-day-old maize seedlings, which were treated with CAPP as grains, was not that high. Prolonged exposure to nitrogen plasma led to a reduction (N90) or complete inhibition (N120–300) of germination, which could be due to excessive oxidative stress and the inability of the antioxidant system to buffer it. A slight increase in DNA damage caused by CAPP treatment was detected, but mostly for longer treatment times. There was also a noticeable level of oxidative DNA damage, especially for ambient air-generated CAPP, however, these were primary DNA damage and cells are capable repairing them. We also found that HSF17 is probably not needed for the regulation of HSPs expression in response to CAPP treatment. On the other hand, the expression of HSP101 and HSP70 genes was induced by CAPP treatment, mostly at the first 48 h after sowing, which suggested that the quick action of molecular chaperones is needed shortly after CAPP treatment.

## Figures and Tables

**Table 1:** Impact of different Cold Atmospheric Pressure Plasma (CAPP) applications on growth parameters of *Zea mays* L. cv. Ronaldinio. Grains were pre-treated with different variants of CAPP (C—control/untreated maize grains; O30–O300: grains treated with plasma generated in an oxygen atmosphere for 30, 60, 90, 120, 180 or 300 s; A30–A300: maize grains treated with plasma generated in ambient air for 30, 60, 90, 120, 180 or 300 s; N30–N300: maize grains treated with plasma generated in a nitrogen atmosphere for 30 and 60 s). Values in table represent means of at least 4 independent experiments (one experiment represents 10–20 measurement data) ± SD according to LSD ANOVA test. Different letters indicate significant difference at *p* < 0.05. Indexes were calculated according to Abdul-Baki and Anderson [56].

CAPP Application (s)	Germination Potential (%)	Germination Index (%)	Grain Vitality Index (%)	Seedling Vitality Index (%)	Seedling Length Index (%)
C (0)	96.9 ± 3.52 f	10.84 ± 1.64 jk	158.1 ± 23.9 fgh	0.34 ± 0.08 ef	172.8 ± 20.5 ef
O30	94.0 ± 8.48 f	8.66 ± 1.88 ghi	205.4 ± 18.5 i	0.34 ± 0.09 ef	189.5 ± 41.2 f
O60	92.3 ± 6.32 f	11.66 ± 0.38 k	124.4 ±2 9.7 cde	0.29 ± 0.11 def	154.2 ± 34.9 def
O90	78.7 ± 12.3 ef	7.00 ± 0.05 efgh	152.9 ± 24.0 efgh	0.27 ± 0.04 cde	136.0 ± 1.0 cde
O120	64.2 ± 5.83 de	7.56 ± 0.79 fgh	92.3 ± 4.7 bc	0.23 ± 0.01 bcde	107.3 ± 11.3 bc
O180	59.5 ± 7.77 bcde	7.00 ± 0.35 efgh	78.5 ± 26.8 b	0.14 ± 0.03 ab	98.1 ± 33.5 bc
O300	58.0 ± 2.82 bcde	4.83 ± 0.23 bcde	73.9 ± 21.2 ab	0.08 ± 0.02 a	90.8 ± 23.6 bc
A30	95.0 ± 7.07 f	8.66 ± 0.47 ghi	189. 6 ± 14.1 hi	0.29 ± 0.06 def	163.8 ± 17.3 ef
A60	93.0 ± 4.35 f	10.75 ± 1.75 ijk	145.6 ± 28.8 ef	0.31 ± 0.09 def	182.1 ± 36.0 f
A90	67.0 ± 4.24 abc	6.16 ± 1.17 cdef	139.1 ± 8.8 def	0.29 ± 0.02 def	113.3 ± 3.7 bcd
A120	68.0 ± 2.82 cde	5.50 ± 0.71 abcd	96.6 ± 4.1 bcd	0.22 ± 0.01 bcd	96.3 ± 5.1 bc
A180	56.7 ± 14.52 abcd	4.40 ± 1.12 abc	94.6 ± 2.3 bc	0.21 ± 0.07 bcd	87.0 ± 12.1 ab
A300	36.2 ± 5.44 ab	2.91 ± 0.87 ab	38.0 ± 5.2 a	0.17 ± 0.01 abc	49.3 ± 6.9 a
N30	98.0 ± 2.82 f	9.00 ± 1.41 hij	188.0 ± 5.4 ghi	0.35 ± 0.05 ef	172.7 ± 27.1 ef
N60	87.2 ± 6.31 f	6.66 ± 1.52 defg	148.4 ± 28.3 efg	0.39 ± 0.11 f	182.5 ± 30.2 f
N90	28.0 ± 0.50 a	2.33 ± 0.23 a	–	–	–
N120	–	–	–	–	–
N180	–	–	–	–	–
N300	–	–	–	–	–

**Table 2:** Sequences of primers for chosen heat shock protein genes.

Gene	Primer	Primer Sequence (5′->3′)
*HSP101* (NM_001111465.2)	HSP101_F	CGAGGTACATCATGGGTCGG
	HSP101_R	GGCTTTACTGGCCTTGTCCT
*HSP70* (NM_001196236.1)	HSP70_F	AGCTTGAGGGGATCTGCAAC
	HSP70_R	CCTCACCAAACCAAACCTCG
*HSF17* (MK736813.1)	HSF17_F	GCCTGATTGGGCCACATGAT
	HSF17_R	CAGGGCTATCGTTCTCCTCG

**Table 3:** Sequences of primers for chosen reference genes.

Gene	Primer	Primer Sequence (5′->3′)
*EF1A* (NM_001112117.2)	EF1α_F	TGGGCCTACTGGTCTTACTACTGA
	EF1α_R	ACATACCCACGCTTCAGATCCT
*TUB B* (NM_001112218.1)	TUB β_F	CTACCTCACGGCATCTGCTATGT
	TUB β_R	GTCACACACACTCGACTTCACG
